# Analysis of Furan in Red Pepper Powder Treated by Three Methods-Boiling, Roasting, and Frying

**DOI:** 10.3389/fnut.2022.888779

**Published:** 2022-05-06

**Authors:** Sookyoung Kim, Haeun Lee, Kwang-Geun Lee

**Affiliations:** Department of Food Science and Biotechnology, Dongguk University-Seoul, Seoul, South Korea

**Keywords:** furan, red pepper, cooking, kinetics, GC-MS

## Abstract

In this study, furan analysis was conducted on dried red pepper powder treated by three cooking methods (boiling, roasting, and frying). A total of 144 samples were prepared and their furan levels were analysed using automated solid-phase micro-extraction gas chromatography-mass spectrometry. The furan concentration in boiled soup ranged from 1.26 to 4.65 ng/g, and from 7.37 to 27.68 ng/g for boiled red pepper samples. For the roasting method, a furan concentration between 6.66 and 761.37 ng/g was detected. For the frying method, the furan level of edible oils ranged from 3.93 to 125.88 ng/g, and a concentration ranging from 4.88 to 234.52 ng/g was detected for the fried red pepper samples. The cooking method using edible oil obtained a higher furan concentration than the water-based method. Samples using corn germ oil (linoleic acid-rich oil) obtained the highest furan concentration among the four edible oils. In all cooking methods, the higher the heating temperature and time, the higher the furan concentration detected. A kinetic study was conducted using the roasting model system and the apparent activation energy was 60.5 kJ/mol. The results of this study could be useful as a database for furan concentration in dried red pepper powder according to various cooking methods.

## Introduction

Dried red pepper powder (*Capsicum annum L*.), globally used as a natural flavouring and colouring agent, is one of the most popular spices because of its unique pungency (1). Approximately 1,990,000 ha of land worldwide and 31,146 ha in South Korea are used to grow red peppers (2). Dried red pepper powder is a major spice in traditional Korean foods, such as gochujang and kimchi. The annual consumption of dried red pepper powder in Korea is 2.5 to3.5 kg per capita (3), and the intake level of capsaicinoids and carotenoids derived from red pepper is significantly high in Korean dietary culture. Most harvested fresh red peppers are processed into powder by a drying process. As cooked foods using dried red pepper powder could be a major contributor to furan exposure, according to previous research, dried red pepper powder may release furan precursors during cooking (4, 5).

Thermal processing of food is a dynamic process involving heat and mass transfer. This results in several physical and chemical changes, including not only sensory characteristic changes but also the formation of safety-related compounds (6–8). Among the process-induced compounds, furan is known to be produced by the degradation and rearrangement of carbohydrates, amino acids, polyunsaturated fatty acids (PUFAs), ascorbic acid, and carotenoids after high-temperature treatment (9). Because dried red pepper powder contains approximately 17.4% fatty acids and red carotenoids, particularly capsaicin (4), furan is very likely to be formed during heat treatment processing.

Furan is classified not only as “reasonably anticipated to be a human carcinogen” according to the U.S. Department of Health and Human Services but also “possibly carcinogenic to humans” (Group 2B) by the International Agency for Research on Cancer (10). Moreover, the U.S. Food and Drug Administration (FDA) reported that relatively high levels of furan could be found in thermally processed foods such as jarred and canned foods (11). The formation of furan, typically produced by heat treatment, has been investigated from various model systems under several temperature conditions (12–14). However, few studies on furan in cooked foods other than jarred or canned foods have been published (15–17).

Foods are complex systems in which chemical compounds are involved in many simultaneous steps. Consequently, it is effective to establish model systems that are widely used to understand the fate of reactions. The development of model systems derived from kinetic data is a useful tool for estimating kinetic parameters and understanding the reaction mechanism (9). To investigate the furan formation occurring in foods, we established model systems based on three cooking methods such as boiling, roasting, and frying.

This study aims to analyse the furan concentration in dried red pepper powder treated by three cooking methods such as boiling, roasting, and frying. In addition, the kinetics of furan in roasted dried red pepper powder and the effect of fatty acid composition of the oil in which the powder was fried was investigated. These findings would be useful for establishing and controlling thermal processing conditions in various foods that include dried red pepper powder.

## Materials and Methods

### Chemical Reagents and Materials

Furan (99% purity) and d4-furan (98% purity) were purchased from Sigma-Aldrich Corporation (St. Louis, MO, United States). HPLC-grade methanol, water, hexane, and acetone were purchased from J.T. Baker (Phillipsburg, NJ, United States). Sodium chloride was supplied by Samchun Pure Chemical Co. (Seoul, South Korea). For furan analysis, the internal standard was prepared with a working solution of d4-furan at a concentration of 1 μg/mL in HPLC-grade water. The whole solution was stored at 4°C until use.

The Korean red peppers powder (*Capsicum annuum* L., Kumdang) were produced and processed at Hansaeng Co. (Seocheon, South Korea). According to the processing manual provided by Hansaeng Co., the naturally sun-dried red peppers were cleaned with air and the pericarp was removed. After grinding, metal dust was removed using a metal detector. Ultraviolet (UV) light was used for sterilisation for 3 min. The red pepper powder was packaged and stored at room temperature before delivery. The edible oils (palm oil, soybean oil, olive oil, and corn germ oil) used in this study were purchased from a commercial market located in Goyang, South Korea.

### Cooking Methods of Dried Red Pepper Powder for Furan Analysis

The cooking methods used in this study were divided into boiling, roasting, and frying as shown in Supplementary Tables 1, 2. As a control, untreated dried red pepper was used. For the boiling, 100 mL of distilled water and 5 g of dried red pepper powder were used. Boiling was conducted at 80 and 100°C, for 20 s, 5, 10, 15, 20, 25, and 30 min using a water bath (Daihan Scientific Co., Seoul, South Korea). Roasting was performed using a temperature-controlled oven (L9282, Convex Co., Seoul, South Korea) at 60, 80, 100, 120, 150, and 180°C for 1, 3, 5, 10, 15, 20, and 25 min. In the oven samples were placed on glass plate. Frying was carried out with 50 g of dried red pepper powder with 500 mL of oil using an electric deep fryer (SERIE F61-M, Zhejiang Shaoxing Co., Shaoxing, China) at 80, 100, 130, and 170°C for 20 s, 5, 10, 15, and 20 min. The solid part (red pepper) was separated from oil and water after frying and boiling by cooking sieve.

Four edible oils (palm oil, olive oil, soybean oil, and corn germ oil) were divided into three types according to fatty acid composition and used in the frying method. Palm oil was used as a saturated fatty acid-rich oil, olive oil as an oleic acid-rich oil, and soybean and corn germ oil as a linoleic acid-rich oil (18). To investigate the effects of fatty acid composition on furan formation, dried red pepper powder was fried at 140°C for 5, 10, 15, and 20 min, following a previous study with modifications (19).

### Analysis of Furan in Boiled, Roasted, and Fried Dried Red Pepper Powder by Automated SPME-GC/MS

Regarding to the furan analysis the analytical method was carried out based on the previous reports (6, 9, 13, 14). The furan (99% purity) and d4-furan (98% purity) solutions were refrigerated at 4°C and –18°C, respectively. The stock and intermediate solutions were prepared using HPLC-grade methanol at a concentration of 10,000 and 100 μg/mL, respectively. The working solution was prepared by serial dilution using HPLC-grade water at a concentration of 1 μg/mL. To make the stock solution, 100 μg of each standard solution was added to a 20 mL volumetric flask independently and made up to 10 mg (10,000 μg/mL) with methanol. To make the intermediate solution, 100 μg of each stock solution was transferred to a 20 mL volumetric flask and made up to 10 mL (100 μg/mL) with methanol. The working solution was prepared by diluting the intermediate solution with HPLC-grade water (1 μg/mL). All the solutions were sealed in 20 mL vials with silicone-PTFE septa and aluminium caps. The stock and intermediate solutions were stored at 4°C until use, and the working solution was prepared each morning before analysis.

For furan analysis, 9 mL of HPLC-grade water was added to 20 mL vials containing 1 g of solid samples. For liquid samples, 5 mL of HPLC-grade water was added to 20 mL vials containing 5 mL of the sample, and 10 μg of internal standard (d4-furan, 1 μg/mL) was added to each vial. For oil-containing samples, the method described by Juániz, Zocco (20) was modified as follows. After frying 50 g of dried pepper powder with 500 mL of oil, the samples were kept in an icebox to avoid furan loss. Immediately, 2 g of fried red pepper powder was transferred to 20 mL vials containing 3 g of NaCl and 5 mL of HPLC-grade water. For the oil samples, 2 mL of oil was transferred to 20 mL vials containing 3 g of NaCl. Additionally, 10 and 5 μL of d4-furan working solution (1 μg/mL) was added as the internal standard for the fried red pepper and oil, respectively. Each sample was immediately closed and prepared in triplicate.

Agilent Technologies 7820A gas chromatograph (Agilent Technologies, Santa Clara, CA, United States) and Agilent Technologies 5977E mass spectrometer with automated SPME (Multipurpose-Sampler, Gerstel, Germany) were used to analyse the furan concentration. Sample extraction was processed according to the method of previous reports (6, 13). The sample was exposured to Carboxen/Polydimethylsiloxane (CAR/PDMS) fibre (Supelco, Bellefonte, PA, United States) for 20 min. Sample-containing vials were pre-heated while shaking at 300 rpm at 50°C in an automated SPME incubator. After extraction, the SPME fibre was removed from the vial and inserted into a GC injector for desorption for 5 min. Helium (+99.9999%) was used as the carrier gas and the flow rate was 1.5 mL/min. The gas chromatograph was operated in splitless mode with the injector maintained at 250°C. Chromatographic separation was performed on an HP PLOT-Q column (15 m, 0.32 mm I.D., 20 μm film thickness; J&W Scientific, Folsom, CA, United States). The GC oven temperature program was applied as follows: 50°C for 2 min, then increased to 230°C at a rate of 25°C/min. After 14.2 min run time, a post-run at 230°C for 5 min is performed. Furan was quantified using the selected ion monitoring mode (SIM mode, +EI, 70 eV), and *m/z* 68 [M]+ /39 [M_CHO]+ was used for furan, and m/z 72 [M]+ /42 [M_CHO]+ for d4-furan.

### Validation of Furan Analysis

The method was validated by obtaining a linear relationship between the furan concentration and the respective area ratio. The limit of detection (LOD), limit of quantification (LOQ), recovery, and precision (intra- and inter-day) were calculated according to the guidelines provided by AOAC (11, 14, 21). The calibration curve was drawn using eight concentration points (0, 2.5, 5, 25, 150, 250, 500, and 1,500 ng/mL) and linearity was expressed by the coefficient of determination (R^2^). LOD and LOQ were calculated from specific calibration curves. The slope of the calibration curve (m), and the standard deviation of the y-intercept (σ) of the regression lines were used for LOD (3.3 × standard deviation/slope of the calibration curve) and LOQ (10 × standard deviation/slope of the calibration curve) calculation (22). To evaluate the recovery, three concentration points (10, 50, and 100 ng/g) were analysed for 5 days and shown as relative standard deviation (RSD, %). Precision was presented with intra- and inter-day, and also shown as relative standard deviation (RSD, %) at the same concentration points (10, 50, and 100 ng/g).

### Kinetic Parameter Analysis

The kinetic parameters were calculated by Eq. (1), according to Arrhenius’ law.


(1) $$\ln\left(\text{k}\right)=\ln\left(\text{A}\right)-\frac{Ea}{R}\left(\frac{1}{T}\right)\ldots \ldots \ldots$$

When k is the reaction rate constant (mol/L⋅h), Ea is the apparent activation energy (kJ/mol), T is the absolute temperature (K), R is the gas constant (8.314 J/mol), and A is the frequency factor. The apparent activation energy of the furan generation reaction in dried red pepper powder was calculated from the straight regression line, with ln(k) as the *y*-axis and 1/T as the *x*-axis, which was obtained using the least-squares method.

### Statistical Analysis

The experimental design was a completely randomised design with three replicates and is presented as mean ± standard deviation (SD). Each data was analysed using ANOVA and Duncan’s multiple range test to investigate significant differences (*p* < 0.05). Statistical analysis was performed by IBM SPSS Statistics 23 (IBM, Chicago, IL, United States).

## Results and Discussion

### Validation for Furan Analysis

The assay was validated by evaluating the linearity (coefficient of determination; R^2^), limit of detection (LOD), limit of quantification (LOQ), recovery, and precision (intra- and inter-day) (Supplementary Table 3). Eight different concentrations in water (0, 2.5, 5, 25, 150, 250, 500, and 1,500 ng/mL) were prepared for the standard calibration curve. The regression equation and linearity of the furan concentration were obtained as *y* = 0.1698× – 1.1645 (*R*^2^ = 0.9980) (Supplementary Figure 1). The limit of detection (LOD) and limit of quantification (LOQ) were calculated (0.12 and 0.35 ng/mL, respectively). According to previous studies analysing furan concentration in instant noodles, the LOD and LOQ were 0.13 and 0.39 ng/mL, respectively (6). The recovery test was calculated from 98.5 to 101.0%. The precision ranged from 2.01 to 7.79 (RSD%) or intra-day and 0.98 to 2.69 (RSD%) for inter-day.

### Furan Concentration in Dried Red Pepper Powder Prepared by Boiling and Roasting

A total of 104 samples were used to investigate the furan concentration in dried red pepper powder treated by various cooking methods (boiling, roasting, and frying). The furan concentration in the boiled red pepper samples was 1.26 ± 0.07 – 4.65 ± 0.34 ng/g in the soup and 7.37 ± 0.14 – 27.68 ± 1.51 ng/g in the solid sample which is pepper powder recovered from water, as shown in Table 1. In untreated dried red pepper powder the furan level was 4.58 ng/g. As boiling temperature increased (80 to 100°C) furan levels in soup and solid sample were increased up to 112 and 251%, respectively. At the same boiling temperature furan levels of soup were increased up to 73% (80°C) and 118% (100°C) as boiling time increased (20 s to 30 min). In the solid sample at the same boiling temperature furan levels were increased up to 6% (80°C) and 117% (100°C) as boiling time increased (20 s to 30 min).

**TABLE 1:** Furan concentration of boiled red pepper and soup samples.

Type	Temperature (°C)	Time (min)	Furan (ng/g)
Liquid (soup)	80	20 s	1.26 ± 0.07^a^
		5	1.48 ± 0.07^b^
		10	1.74 ± 0.04^c^
		15	1.78 ± 0.04^c^
		20	1.89 ± 0.01^d^
		25	1.94 ± 0.08^d^
		30	2.19 ± 0.04^e^
	100	20 s	2.13 ± 0.08^a^
		5	2.66 ± 0.08^b^
		10	2.81 ± 0.10^b^
		15	3.68 ± 0.24^c^
		20	4.63 ± 0.35^d^
		25	4.25 ± 0.14^d^
		30	4.65 ± 0.34^d^
Solid (pepper powder recovered from water)	80	20 s	7.37 ± 0.14^a^
		5	7.57 ± 0.26^a,b^
		10	7.65 ± 0.17^a,b^
		15	7.83 ± 0.24^b^
		20	7.52 ± 0.22^a,b^
		25	7.39 ± 0.11^a^
		30	7.88 ± 0.23^b^
	100	20 s	12.75 ± 0.84^a^
		5	15.32 ± 0.69^b^
		10	17.45 ± 1.17^c^
		15	14.69 ± 1.06^b^
		20	19.26 ± 0.79^c,d^
		25	21.00 ± 1.16^d^
		30	27.68 ± 1.51^e^

*All values are shown as mean ± S.D. (standard deviation) (n = 3). Lowercase letters (series “a–e”) indicate significant (p < 0.05) differences during boiling dried red pepper powder.*

The furan concentration in the solid sample, pepper powder recovered from water (100°C, 30 min) was 5.95 times higher than that of the soup sample prepared under the same conditions (100°C, 30 min). Overall, an approximately six-fold higher furan content was detected in the solid compared to the soup. This result was similar to that of the previous study, which analysed the furan content in the soup isolated from instant noodles, representing values from 1.02 ± 0.11 to 2.45 ± 0.05 ng/g (6). Regarding to the proportion of spices in various soup samples were reported in the previous studies (23, 24).

The United States Department of Agriculture (USDA) investigated that 100 g of red pepper powder contains approximately 50%(w/w) of carbohydrates, 14%(w/w) of fat, 13%(w/w) of protein, and other nutritional components such as minerals and vitamins (25). As thermal degradation of carbohydrates is the primary contributor to the level of furan in food, the content of dried red pepper powder could influence the level of furan in instant noodle soups (26). Considering that the furan content increased with increasing heat treatment and time (27), it was expected that the furan concentration of boiled soup sample treated at 100°C for 30 min (4.65 ± 0.34 ng/g) was higher than that detected in the instant noodle soup treated at 100°C for 3 min. For the solid type, boiled red pepper samples prepared at 80°C did not show a statistically significant difference (*p* < 0.05). However, in the case of boiled red pepper samples cooked at 100°C, the furan levels significantly increased with increasing time.

The furan concentration in the roasted red pepper samples is shown in Table 2. During the roasting process, several changes occur in the food matrix. In untreated dried red pepper powder the furan level was 4.58 ng/g. In the previous study, furan production was found to be temperature-dependent in hot coffee samples (28). The furan levels in the roasted red pepper samples also increased with increasing temperature and time conditions. The furan levels in roasted red pepper samples ranged from 6.66 ± 0.30 to 761.37 ± 24.60 ng/g. The minimum furan level was detected when roasting was performed at 80°C for 1 min, and the maximum value was detected at 100°C for 30 min. The furan content increased significantly as time increased above 100°C (*p* < 0.05). There was a dramatic increase in furan levels when the roasting time increased from 5 to 10 min, during roasting above 120°C. Compared to untreated dried red pepper powder (6.84 ± 0.16 ng/g), the furan concentration was 1.03 times higher (60°C, 20 min), and 111.31 times higher (180°C, 20 min). The roasting results were used to investigate the kinetic parameters.

**TABLE 2:** Furan concentration of roasted red pepper samples.

Temperature (°C)	Time (min)	Furan (ng/g)	Temperature (°C)	Time (min)	Furan (ng/g)
60	1	6.95 ± 0.07^a,A,B^	120	1	7.41 ± 0.04^a,B,C^
	3	7.30 ± 0.14^c,A,B^		3	7.65 ± 0.20^a,B^
	5	7.33 ± 0.15^c,A,B^		5	8.50 ± 0.18^a,B^
	10	7.19 ± 0.19^b,c,A^		10	13.89 ± 0.89^b,A^
	15	7.61 ± 0.09^d,A^		15	33.05 ± 3.10^c,B^
	20	7.04 ± 0.04^a,b,A^		20	66.45 ± 2.55^d,B^
80	1	6.66 ± 0.30^a,A^	150	1	8.86 ± 0.80^a,D^
	3	6.79 ± 0.15^a,b,A^		3	10.22 ± 0.57^a,C^
	5	7.10 ± 0.09^c,A^		5	17.44 ± 0.19^a,C^
	10	7.01 ± 0.04^b,c,A^		10	121.29 ± 8.04^b,B^
	15	7.67 ± 0.13^d,A^		15	238.23 ± 15.72^c,C^
	20	8.08 ± 0.02^e,A^		20	493.09 ± 53.55^d,C^
100	1	7.45 ± 0.23^a,B,C^	180	1	7.75 ± 0.18^a,C^
	3	7.52 ± 0.31^a,B^		3	9.94 ± 0.56^a,C^
	5	7.84 ± 0.12^a,A,B^		5	20.19 ± 1.02^a,D^
	10	10.32 ± 1.70^b,A^		10	131.88 ± 15.09^b,B^
	15	11.30 ± 0.52^b,A^		15	619.90 ± 11.75^c,D^
	20	13.11 ± 0.48^c,A^		20	761.37 ± 24.60^d,D^

*All values are shown as mean ± S.D. (standard deviation) (n = 3). Lowercase letters (series “a–d”) indicate significant (p < 0.05) differences at same experimental temperature with different heating time. Uppercase letters (series “A–D”) indicate significant (p < 0.05) differences at same heating time with different experimental temperature.*

### Furan Concentration in Fried Red Pepper Samples According to Edible Oils

The furan levels in the soybean oil fried red pepper samples are listed in Table 3. The furan levels ranged from 3.93 ± 0.05 to 125.88 ± 0.55 and 4.88 ± 0.12 to 234.52 ± 15.09 ng/g in the fried soybean oil and the fried red pepper samples, respectively. At the same temperature condition, increasing heating time indicated resulted in higher furan concentration. Compared to the control (untreated dried red pepper powder), the furan concentration of the fried red pepper sample (80°C, 20 min) increased by 123.44%.

**TABLE 3:** Furan concentration of fried red pepper and soybean oil samples.

Type	Temperature (°C)	Time (min)	Furan (ng/g)	Type	Temperature (°C)	Time (min)	Furan (ng/g)
Liquid (oil)	80	20 s	3.93 ± 0.05^a,A^	Solid (pepper powder recovered from water)	80	20 s	4.88 ± 0.12^a,A^
		5	4.31 ± 0.00^b,A^			5	7.41 ± 0.16^b,A^
		10	4.29 ± 0.06^b,A^			10	7.46 ± 0.27^b,A^
		15	4.57 ± 0.11^c,A^			15	7.11 ± 0.12^b,A^
		20	8.58 ± 0.03^d,A^			20	12.69 ± 0.67^c,A^
	100	20 s	4.01 ± 0.10^a,A^		100	20 s	5.50 ± 0.07^a,B^
		5	6.27 ± 0.18^b,B^			5	14.14 ± 1.17^b,B^
		10	9.67 ± 0.11^c,B^			10	19.14 ± 0.96^c,B^
		15	13.20 ± 0.26^d,B^			15	20.78 ± 0.35^d,B^
		20	17.99 ± 0.74^e,B^			20	20.79 ± 0.80^d,A^
	130	20 s	5.09 ± 0.04^a,B^		130	20 s	8.12 ± 0.39^a,C^
		5	22.62 ± 0.66^b,C^			5	47.64 ± 3.00^b,C^
		10	34.02 ± 0.75^c,C^			10	57.86 ± 3.24^d,C^
		15	42.01 ± 1.55^d,C^			15	56.94 ± 1.73^c,d,C^
		20	42.58 ± 0.90^d,C^			20	53.62 ± 0.95^c,B^
	170	20 s	22.53 ± 0.83^a,C^		170	20 s	15.20 ± 0.25^a,D^
		5	60.74 ± 0.70^b,D^			5	103.48 ± 4.69^b,D^
		10	100.73 ± 2.56^c,D^			10	134.89 ± 11.27^c,D^
		15	121.47 ± 2.33^d,D^			15	168.18 ± 3.91^d,D^
		20	125.88 ± 0.55^e,D^			20	234.52 ± 15.09^e,C^

*All values are shown as mean ± S.D. (standard deviation) (n = 3). Lowercase letters (series “a–e”) indicate significant (p < 0.05) differences at same experimental temperature with different heating time. Uppercase letters (series “A–D”) indicate significant (p < 0.05) differences at same heating time with different experimental temperature.*

From the above results, it was found that furan formation in dried red pepper powder increased with increasing heating time and temperature in the different cooking methods. The results of comparing the furan concentration of three cooking methods under the common temperature (80 and 100°C) and time (5, 10, 15, and 20 min) conditions are shown in Supplementary Figures 2, 3. The furan levels of the boiled soup samples showed the lowest values at all time and temperature conditions. At 80°C, the red pepper samples prepared using the three cooking methods revealed similar levels of furan content up to 15 min, and the furan concentration of the fried red pepper samples increased sharply at 20 min. In the case of the oil-fried samples, the furan levels were twice as high as those of the soup (boiling) up to 15 min but increased 4.54 times at 20 min. When cooking at 100°C, the furan levels of the boiled and fried red pepper powder samples were higher than those of roasted. In the case of frying, both fried red pepper and edible oil samples showed a gradual increase with increasing time. From the results obtained at 80 and 100°C, the effect of edible oil is greater than that of water on furan generation during heat based-cooking methods.

During the thermal processes performed by the three cooking methods, furan formation in the dried red pepper powder increased. This result was similar to that of a previous study, according to which thermal degradation of carbohydrates and ascorbic acid and thermal oxidation of polyunsaturated fatty acids (PUFAs) are the main pathways for furan formation in foods (29). However, there is a wide range of cooking conditions between each method, making it difficult to compare furan levels accurately. In addition, the cooking method for each ingredient could vary between menus even for a dish containing the same ingredients (26). Therefore, the roasting model system was used to analyse the kinetic parameters of furan concentration according to thermal capacity.

One of the possible mechanisms of furan generation is from PUFAs, resulting in lipid peroxidation products like 2-alkenal, 4-oxo-alkenal, 4-hydoxy-2-alkenal, and 4-hydroxy-2-butanal (9). In addition, Becalski and Seaman revealed that model systems heated at 118°C for 30 min indicated that only PUFAs, especially linoleic and linolenic acids, can generate furan during heating (21, 30).

From the results above (Supplementary Figures 2, 3), it was revealed that the dried red pepper powder fried with soybean oil contained a higher furan concentration than that cooked with water under the same temperature conditions (80 and 100°C). This means that edible oil affected furan formation more than water. A total of 40 samples were analysed and four types of edible oils were used to investigate how fatty acid composition affects furan production (Table 4). Palm oil was used as saturated fatty acid-rich oil, olive oil as oleic acid-rich oil, and soybean and corn germ oil as linoleic acid-rich oil (18, 31, 32).

**TABLE 4:** Furan concentration of fried edible oil samples and fried red pepper samples.

Temperature (°C)	Oil type	Time (min)	Furan (ng/g)	
			Liquid	Solid
140	Palm oil	control	3.61 ± 0.26^a,A^	4.58 ± 0.07^a,A^
		5	20.09 ± 0.29^b,A^	68.05 ± 3.30^b,B^
		10	30.69 ± 1.00^d,A^	78.54 ± 5.35^c,A,B^
		15	36.40 ± 0.14^e,A^	78.72 ± 2.95^c,A^
		20	27.55 ± 1.03^c,A^	105.39 ± 8.53^d,B^
	Olive oil	control	4.94 ± 0.06^a,C^	4.58 ± 0.07^a,A^
		5	29.93 ± 1.00^b,B^	58.58 ± 6.09^b,A^
		10	31.66 ± 1.36^c,A^	71.06 ± 6.64^c,A^
		15	39.58 ± 1.68^e,A^	84.98 ± 7.12^d,A^
		20	35.23 ± 2.19^d,B^	78.98 ± 5.57^c,d,A^
	Soybean oil	control	3.84 ± 0.11^a,A,B^	4.58 ± 0.07^a,A^
		5	33.84 ± 2.26^b,D^	71.34 ± 4.73^b,B^
		10	42.00 ± 1.64^c,B^	74.35 ± 8.16^b,A^
		15	47.06 ± 1.24^d,B^	86.25 ± 1.92^c,A^
		20	48.28 ± 3.41^d,C^	88.33 ± 5.56^c,A^
	Corn-germ oil	control	4.09 ± 0.21^a,B^	4.58 ± 0.07^a,A^
		5	27.95 ± 3.04^b,C^	65.79 ± 3.29^b,A,B^
		10	43.32 ± 3.47^c,B^	87.46 ± 6.14^c,B^
		15	52.50 ± 4.20^d,C^	102.37 ± 7.05^d,B^
		20	69.87 ± 6.03^e,D^	103.61 ± 7.20^d,B^

*All values are shown as mean ± S.D. (standard deviation) (n = 3). Lowercase letters in each type (series “a–e”) indicate significant (p < 0.05) differences at same oil type with different heating time. Uppercase letters in each type (series “A–D”) indicate significant (p < 0.05) differences at same heating time with different oil types. Control means that the furan level in untreated red pepper powder.*

After frying, the results showed that increasing the heating time increased the furan concentration regardless of the sample and edible oil types. In the case of fresh oil, furan levels ranged from 3.61 ng/g (palm oil) to 4.94 ng/g (olive oil). The highest furan level was observed in corn germ oil, followed by soybean oil. Furthermore, the results revealed that both corn germ oil and soybean oil (linoleic acid-rich oils) produced a higher furan concentration compared to olive oil (oleic acid-rich oil) and palm oil (saturated fatty acid-rich oil) under all-time conditions. This is similar to the results reported in earlier reports of furan formation from PUFAs upon thermal treatments (21, 33). The furan concentration in the edible oil samples, fried with olive and palm oil, tended to decrease when changing from 15 to 20 min. However, corn germ oil and soybean oil, which are linoleic acid-rich oils, showed an increase in furan concentrations with increasing frying time from 15 to 20 min. In the solid type, the highest furan concentration was 105.39 ng/g observed in the sample cooked with palm oil for 20 min. The furan levels of the solid samples fried with palm oil increased rapidly from 15 to 20 min. The results from frying with the four edible oils were similar up to 5 min; however, the furan levels obtained when using corn germ oil were higher than the others after 10 min (*p* < 0.05).

Comparing the samples using corn germ oil (140°C, 20 min) in which the highest furan concentration was detected, a furan content 1.48 times higher than that of the oil type was detected in the solid type. The results revealed that oil types containing various fatty acids could influence furan production when frying dried red pepper powder. Red pepper powder fried in linoleic acid-rich edible oil such as corn germ oil had higher furan level compared to other edible oils.

### Kinetic Study in Dried Red Pepper Powder With the Roasting Model System

**FIGURE 1:**
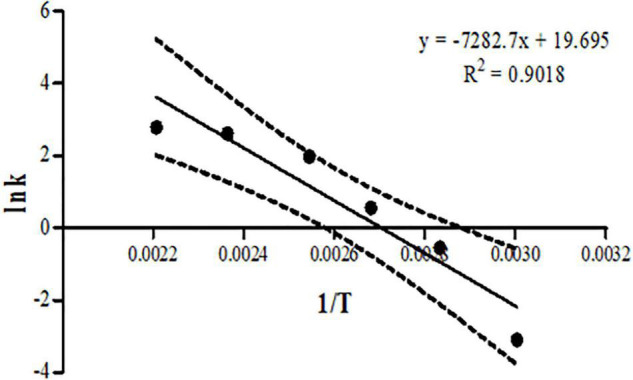
Linear regression of Arrhenius equation in dried red pepper samples.

The results of the furan concentration obtained by the roasting method were used for the kinetic analysis. The activation energy was obtained by substituting the result into the Arrhenius equation. The Arrhenius equation was induced by analysing the samples manufactured by the roasting model system using the thermal instrument (L9282, Convex Co., Seoul, South Korea) at six temperatures and six time intervals. The slope, calculated by performing linear regression analysis for each temperature, was used to calculate the activation energy. The activation energy obtained from the Arrhenius formula was 60.548 kJ/mol, as shown in Figure 1. In the previous study the activation energy for the furan formation in roasted various nuts such as almonds, peanuts, and cashew nuts were 62 to 125 kJ/mol (9). Using the kinetic parameters, it was possible to analyse the amount of reaction according to temperature conditions (34).

Kinetic studies and mathematical models on quality changes of foods are essential in the proper design of thermal treatments to ensure consumer satisfaction (35). Kinetic parameters can be used to predict the influence of heat capacity on furan formation. Foods are complex matrices, which are not composed of a single component. In addition, furan can be formed through various mechanisms; therefore, it is difficult to predict the response path accurately. However, it is possible to predict furan levels in roasted red pepper powder based on temperature conditions via kinetic parameters. Therefore, this study could provide a quality database for dried red pepper powder during thermal processing by designating furan as a possibly produced compound during heat treatment.

## Conclusion

In this study, we analysed the furan concentration of dried red pepper powder formed during the cooking process. A total of 144 samples were analysed, and the samples prepared by the roasting method were used in the kinetic study to calculate the activation energy. The cooking method using edible oil obtained a higher furan concentration than the water-based cooking method. The samples fried with linoleic acid-rich oil revealed the highest furan concentrations among the three types of edible oil. In all cooking methods, the higher the heating temperature and time, the higher the furan concentration detected. As boiling temperature increased furan levels in the sample were increased up to 251% and an approximately six-fold higher furan content was detected in the solid compared to the soup. The furan levels in roasted and fried red pepper samples were up to 761.37 and 234.52 ng/g, respectively. The activation energy in the roasting model system calculated by the Arrhenius equation was 60.5 kJ/mol. The results of this study would be useful as a database for furan concentration in dried red pepper powder according to various cooking methods. In addition, it could be used to predict furan formation using kinetic parameters.

## Data Availability Statement

The original contributions presented in the study are included in the article/Supplementary Material, further inquiries can be directed to the corresponding author.

## Author Contributions

SK: formal analysis, investigation, and methodology. HL: formal analysis. K-GL: supervision, validation, investigation, and project administration. All authors contributed to the article and approved the submitted version.

## Conflict of Interest

The authors declare that the research was conducted in the absence of any commercial or financial relationships that could be construed as a potential conflict of interest.

## Publisher’s Note

All claims expressed in this article are solely those of the authors and do not necessarily represent those of their affiliated organizations, or those of the publisher, the editors and the reviewers. Any product that may be evaluated in this article, or claim that may be made by its manufacturer, is not guaranteed or endorsed by the publisher.
