# Supplementary material for: Analysis of Furan in Red Pepper Powder Treated by Three Methods-Boiling, Roasting, and Frying
Source: Front Nutr. 2022 May 6;9:888779. doi: 10.3389/fnut.2022.888779 (PMC9149621; doi:10.3389/fnut.2022.888779)
Supplement: Supplementary file 1 [file Data_Sheet_1.docx]

**Supplementary Table 1. Preparation of dried red pepper samples according to cooking methods**

| **Cooking method** | **Sample type** | **Cooking temperature (℃)** | **Cooking time (min)** |
| --- | --- | --- | --- |
| Boiling | Solid | 80 | 20 sec / 5 / 10 / 15 / 20 / 25 / 30 |
|  |  | 100 | 20 sec / 5 / 10 / 15 / 20 / 25 / 30 |
|  | Liquid (soup) | 80 | 20 sec / 5 / 10 / 15 / 20 / 25 / 30 |
|  |  | 100 | 20 sec / 5 / 10 / 15 / 20 / 25 / 30 |
| Roasting | Solid | 60 | 1 / 3 / 5 / 10 / 15 / 20 |
|  |  | 80 | 1 / 3 / 5 / 10 / 15 / 20 |
|  |  | 100 | 1 / 3 / 5 / 10 / 15 / 20 |
|  |  | 120 | 1 / 3 / 5 / 10 / 15 / 20 |
|  |  | 150 | 1 / 3 / 5 / 10 / 15 / 20 |
|  |  | 180 | 1 / 3 / 5 / 10 / 15 / 20 |
| Frying | Solid | 80 | 20 sec / 5 / 10 / 15 / 20 |
|  |  | 100 | 20 sec / 5 / 10 / 15 / 20 |
|  |  | 130 | 20 sec / 5 / 10 / 15 / 20 |
|  |  | 170 | 20 sec / 5 / 10 / 15 / 20 |
|  | Liquid (oil) | 80 | 20 sec / 5 / 10 / 15 / 20 |
|  |  | 100 | 20 sec / 5 / 10 / 15 / 20 |
|  |  | 130 | 20 sec / 5 / 10 / 15 / 20 |
|  |  | 170 | 20 sec / 5 / 10 / 15 / 20 |
| **Total** | | | **104** |

**Supplementary Table 2. Preparation of fried red pepper samples according to edible oils**

| **Frying temperature (℃)** | **Sample type** | **Edible oil type** | **Frying time (min)** |
| --- | --- | --- | --- |
| 140 | Liquid (oil) | Palm oil | Control / 5 / 10 / 15 / 20 |
|  |  | Olive oil | Control / 5 / 10 / 15 / 20 |
|  |  | Soybean oil | Control / 5 / 10 / 15 / 20 |
|  |  | Corn-germ oil | Control / 5 / 10 / 15 / 20 |
|  | Solid | Palm oil | Control / 5 / 10 / 15 / 20 |
|  |  | Olive oil | Control / 5 / 10 / 15 / 20 |
|  |  | Soybean oil | Control / 5 / 10 / 15 / 20 |
|  |  | Corn-germ oil | Control / 5 / 10 / 15 / 20 |
| **Total** | | | **40** |

1. Control means that the furan level in untreated red pepper powder.

**Supplementary Table 3. Validation for furan analysis in dried red pepper powder using SPME-GC/MS**

| Calibration curve^a^ | | LOD^b^ (ng/mL) | LOQ^c^ (ng/mL) |
| --- | --- | --- | --- |
| Equation | R^2^ |  |  |
| y = 0.1698x – 1.1645 | 0.9980 | 0.12 | 0.35 |
| Concentration (ng/mL) | Recovery^d^ (%) | Intra-day (RSD, %) | Inter-day (RSD, %) |
| 1 | 115.8 | 2.01 | 2.69 |
| 50 | 98.5 | 7.79 | 2.35 |
| 100 | 101.0 | 2.20 | 0.98 |
| Calibration curve is analyzed in triplicate, LOD and LOQ were conducted seven times. | | | |
| ^a^ Range of calibration curve was measured at 8 points: 0, 2.5, 5, 25, 150, 250, 500 and 1500ng/mL in water | | | |
| ^b^LOD = 3.14 * SD/slope (ng/mL), ^c^LOQ = 10 * SD/slope (ng/mL) | | | |
| ^d^Recovery (%) = (volume of furan in spiked standard solution – volume of furan in standard solution) × 100 / volume of spiking furan | | | |

**Supplementary Table 4. SPME-GC/MS conditions for furan analysis**

| **Solid phase microextration (SPME)** | |
| --- | --- |
| SPME fiber | 75μm Carboxen/polydimethylsiloxane (CAR/PDMS) |
| **Gas chromatography** | |
| Instrument | Agilent 7820A gas chromatography |
| Carrier gas | 1.5 ml/min helium (constant flow) |
| Column | HP-PLOT Q (15 m × 0.32 mm, 20 μm film, J & W Scientific, Folsom., CA) |
| Inlet temperature (℃) | 250 ℃ |
| Purge flow rate | 50 ml/min for 1 min |
| Oven temperature (℃) | 50 ℃ for 5min |
|  | 25℃/min to 230℃ held for 2 min |
| Injection mode | Splitless mode |
| **Mass spectrometry** | |
| Instrument | Agilent 5977E mass spectrometry detector |
| Fragmentation mode | Electron impact at 70eV |
| SIM | m/z 39, **68** for furan |
|  | m/z 42, **72** for d4 furan |

**Supplementary Figure 1. Calibration curve of furan using SPME-GC/M**

**Supplementary Figure 2. Comparison of furan levels in cooked (boiling, roasting and frying) red pepper, soup and oil samples at 80** ℃

**Supplementary Figure 3. Comparison of furan levels in cooked (boiling, roasting and frying) red pepper, soup and oil samples at 100** ℃
